# Prevalence and Risk Factors of Asymptomatic Peripheral Arterial Disease in Patients with COPD in Taiwan

**DOI:** 10.1371/journal.pone.0064714

**Published:** 2013-05-22

**Authors:** Ming-Shian Lin, Kun-Yen Hsu, Yi-Jen Chen, Cheng-Ren Chen, Chuan-Mu Chen, Wei Chen

**Affiliations:** 1 Division of Pulmonary and Critical Care Medicine, Chia-Yi Christian Hospital, Chiayi, Taiwan; 2 Department of Respiratory Care, Chang Gung University of Science and Technology, Chiayi Campus, Taichung, Taiwan; 3 Department of Respiratory Therapy, China Medical University, Taichung, Taiwan; 4 Department of Life Science, National Chung Hsing University, Taichung, Taiwan; University of Leicester, United Kingdom

## Abstract

**Aim:**

Chronic obstructive pulmonary disease (COPD) is an independent risk factor for cardiovascular morbidity and mortality. The aim of this study was to determine the prevalence of asymptomatic peripheral arterial disease (PAD) and the associated risk factors for patients with COPD.

**Methods:**

This prospective cross-sectional study enrolled 427 COPD patients (mean age: 70.0 years) without PAD symptoms consecutively. Demographic data, lung function and cardiovascular risk factors were recorded. The ankle-brachial index (ABI) was used to detect PAD (ABI<0.90).

**Results:**

The overall prevalence of asymptomatic PAD in the COPD patients was 8% (2.5% in the younger participants (<65 years of age, n = 118) and 10% in the elderly participants (≥65 years of age, n = 309). The COPD patients with asymptomatic PAD had a significantly higher rate of hyperlipidemia (47.1% vs. 10.4%) and hypertension (79.4% vs. 45.8%) than those without asymptomatic PAD (p<0.05). There was no significant difference in lung function (forced vital capacity and forced expiratory volume in one second) between the two groups. In multivariate logistic regression, hyperlipidemia was the strongest independent factor for PAD (odds ratio (OR): 6.89, p<0.005), followed by old age (OR: 4.80), hypertension (OR: 3.39) and smoking burden (pack-years, OR: 1.02).

**Conclusions:**

The prevalence of asymptomatic PAD among COPD patients in Taiwan is lower than in Western countries. Hyperlipidemia, old age, hypertension, and smoking burden were the associated cardiovascular risk factors. However, there was no association between lung function and PAD in the COPD patients.

## Introduction

Chronic obstructive pulmonary disease (COPD) is a leading cause of disability and death worldwide, with reported prevalence rates between 5% and 13% [1]–[3]. By 2030, COPD is expected to represent the third leading cause of death in middle-income countries [4]. Smoking is a major cause of COPD, as smoking-induced inflammation causes vascular endothelial damage via oxidative stress [5]. Moreover, COPD has been proven to be an independent factor for systemic inflammation [6], [7]. Both of these factors can contribute to the development of atherosclerotic processes through a common pathway involving oxidative stress [8]–[10]. Therefore, COPD is an independent risk factor for cardiovascular morbidity and mortality [11]–[13].

Peripheral arterial disease (PAD) is an atherosclerotic process that affects non-coronary arteries and often refers to occlusion of the arteries of the lower limbs [14], [15]. The reported prevalence of PAD in COPD patients ranges widely, from 81.4% in a French study [16] to around 30 to 40% in studies from Israel and Spain [17], [18], both of which found that COPD patients with PAD had worse lung function [17], [18]. However, there are currently no studies regarding the prevalence and risk factors of PAD in patients with COPD in Asia.

The primary end point of this study was to determine the prevalence of low ankle-brachial index (ABI<0.9) in COPD patients in Taiwan. The secondary end point was to evaluate the risk factors for PAD and further to investigate whether PAD was associated with worse lung function.

## Materials and Methods

### Study design

A cross-sectional study design was used to examine the baseline data (collected from January 2011 to January 2013) among the participants enrolled in the CMPICO study (Case Management Program and Integrated Care for Patients with Chronic Obstructive Pulmonary Disease). The study was conducted at the outpatient department of the Division of Pulmonary and Critical Care Medicine, Ditmanson Medical Foundation Chia-Yi Christian Hospital, which is a 1,000-bed community-based teaching hospital in Chiayi, Taiwan. The Institutional Review Board of Ditmanson Medical Foundation Chia-Yi Christian Hospital approved the study. All the enrolled patients were kindly requested by the study team to participate in the study. Most of the enrolled individuals provided written informed consent, except a few participants provided verbal consent due to illiteracy. However, our study team member would explain the study design thoroughly for those who were unable to write or read. Eventually, they would sign by their fingerprints in the consent instead.

### Subjects

Patients who had respiratory complaints and then underwent pulmonary function tests at the suggestion of their chest physician were asked to volunteer for PAD screening. All of the patients were free of obvious symptoms of PAD such as intermittent claudication, foot ulcers or pain on walking. In total, 2022 subjects completed the study, of whom we recruited those who had a clinical diagnosis of COPD and were current or former smokers with at least a 10-pack-year history. Eligible patients were 45 to 89 years of age with a post-bronchodilator forced expiratory volume in 1 second (FEV_1_) to forced vital capacity (FVC) of less than 0.7 [19]. Patients with a history of bronchial asthma and other structural lung diseases (such as lung cancer, bronchiectasis, fibrotic lung) were excluded. A chest physician reviewed all of the patients' chest radiographs carefully. A total of 427 patients (97.7% male) with a mean age of 70.0±9.5 years met the inclusion criteria and were enrolled. Once a participant was diagnosed as having asymptomatic PAD, they were referred to the Department of Cardiovascular Surgery for further management. Lifestyle changes (such as smoking cessation and exercise) and pharmacotherapy (for hypertension, diabetes and hyperlipidemia) would then be suggested.

### Data collection

Interviews were conducted by trained nursing staff who used a well-established questionnaire to collect demographic information of the study participants including date of birth, sex, smoking status, and personal medical history. Body weight, height, and blood pressure (BP) were measured for each participant.

Body mass index (BMI) was calculated as weight (kg) divided by the square of the height (m). A BMI<25 was defined as normal weight, from 25 to 30 as overweight, and >30 as obese. The subjects self-reported their medical history including previous hypertension, hyperlipidemia and diabetes mellitus. The participants were considered to be hypertensive if they had a previous diagnosis of hypertension, or if they were on treatment or had a systolic BP (SBP) >140 mmHg or a diastolic BP (DBP) >90 mmHg. The participants were considered to have hyperlipidemia if they had a previous diagnosis of hypercholesterolemia or hypertriglyceridemia, or if they were under treatment or had a fasting cholesterol level of >240 mg/dL or triglycerides >150 mg/dL. Diabetes was defined as a history of diabetes, diabetes treatment, or fasting glucose level >126 mg/dL. Smoking status was evaluated by a self-reported questionnaire. The nursing staff confirmed the medical history by reviewing the participants' medical charts. The severity of COPD was based on the Global Initiative for Chronic Obstructive Pulmonary Disease (GOLD) guidelines [20].

### Measurement of ABI

Ankle and brachial pressures were measured using volume-plethysmographic apparatus (VaSera® VS-1000, Fukuda Denshi, Tokyo, Japan). In addition to taking recordings of the limbs, electrocardiography and mechano-cardiograms were simultaneously recorded by attaching blood pressure cuffs with a tonometric sensor to the upper arm and ankle. The ABI was determined as the ratio of ankle SBP to brachial SBP, with the brachial pressure being measured in the left arm, and the ankle pressure in both the left and right sides with subjects in the supine position. The lowest value of the ABI was used in the analysis. The ABI procedure took approximately 10 minutes to perform [21], and pulse wave velocity (PWV) was measured at the same time. The measurements were carried out twice and averaged for analysis. Participants with an ABI score of <0.90 were defined as having PAD, and those with an ABI score of 0.90 and above as not having PAD [22]. An ABI>1.4 is suggestive of calcified arteries and is associated with aortic stiffness [23], and the pathology may be different between an ABI<0.9 and ABI>1.4. Therefore, only an ABI<0.9 was used as the diagnosis of asymptomatic PAD. In addition, none of the COPD patients had an ABI>1.4 in this study.

### Statistical analysis

Data were analyzed using SPSS statistical software for Windows version 17 (SPSS Inc., Chicago, IL, USA). Continuous data were expressed as mean ± standard deviation. The Student's *t*-test was used when data were normally distributed; otherwise, the non-parametric Mann-Whitney *U*-test was used. Categorical data were compared using the chi-square test if the observed numbers in all categories were larger than 5; otherwise, Fisher's exact test was used. Factors independently associated with the development of PAD were explored using a multiple logistic regression model by forward stepwise analysis.

## Results

Of the 427 participants, 34 (8%) were diagnosed to have asymptomatic PAD. The most common cardiovascular comorbidity was hypertension (n = 207, 48.5%), followed by diabetes mellitus (n = 78, 18.3%), and hyperlipidemia (n = 57, 13.3%). All the enrolled patients had a history of smoking (at least 10 pack-years), including current (n = 220, 51.5%) and former (n = 207, 48.5%) smokers. The mean pack-years of smoking were 54.6±33. Sixty-four patients (15%) had a significant reversibility of short-acting bronchodilators. There was no patient with severe respiratory failure in our study. Ninety-five patients (22.2%) were overweight, 16 (3.7%) were obese.

### Demographic differences between COPD patients with and without asymptomatic PAD

As shown in Table 1, the COPD patients with asymptomatic PAD were significantly older and shorter in height than the COPD patients without asymptomatic PAD (*p*<0.05). With regards to cardiovascular risk factors, a significantly higher proportion of the COPD patients with asymptomatic PAD had hyperlipidemia (47.1% vs. 10.4%), and hypertension (79.4% vs. 45.8%) than the COPD patients without asymptomatic PAD. However, there were no significant differences in terms of PWV, lung function and GOLD stage between the two groups. The age and co-morbidities between the different GOLD grades were shown in Table 2. All the cardiovascular risks had no significance difference between the four stages.

**Table 1:** Demographic data of the COPD patients with and without PAD.

	All (n = 427)	PAD (−) n = 393	PAD (+) n = 34
Age (years)	70.0±9.5 (45∼89)	69.5±9.5	76.1±7.5*
Gender: male	417 (97.7%)	383 (97.5%)	34 (100.0%)
Height (cm)	162.2±6.0	162.4±6.1	160.1±4.9*
BW (kg)	60.8±10.5	60.8±10.5	60.0±11.1
BMI	23.0±3.5	23.0±3.5	23.3±3.6
Normal	316 (74.0%)	292 (74.3%)	24 (70.6%)
Overweight	95 (22.2%)	88 (22.4%)	7 (20.6%)
Obese	16 (3.7%)	13 (3.3%)	3 (8.8%)
Smoker	427 (100.0%)	393 (100.0%)	34 (100.0%)
Pack-years	54.6±33	52.8±30	75.2±47*
Current	220 (51.5%)	204 (51.9%)	16 (47.1%)
Former	207 (48.5%)	189 (48.1%)	18 (52.9%)
DM	78 (18.3%)	68 (17.3%)	10 (29.4%)
Hypertension	207 (48.5%)	180 (45.8%)	27 (79.4%)*
Hyperlipidemia	57 (13.3%)	41 (10.4%)	16 (47.1%)*
Right PWV	16.4±3.9	16.3±3.8	16.0±5.4
Left PWV	16.3±4.2	16.3±3.9	16.7±6.5
ABI	1.06±0.1	1.08±0.1	0.83±0.1*
Lung function			
Pre-bronchodilator			
FEV_1_ (L)	1.15±0.48	1.17±0.48	1.02±0.35
FVC (L)	62.1±17.0	2.02±0.65	1.82±0.49
FEV_1_/FVC (%)	56.8±9.4	56.9±9.5	55.5±8.3
Post-bronchodilator			
FEV_1_ (L)	1.28±0.49	1.28±0.50	1.12±0.36
FEV_1_ (pred %)	51.2±18.0	51.1±18.0	51.8±18.1
FEV_1_/FVC (%)	56.9±9.4	56.9±9.6	56.3±8.1
GOLD stage			
I	28 (6.6%)	26 (6.6%)	2 (5.9%)
II	186 (43.6%)	171 (43.5%)	15 (44.1%)
III	159 (37.2%)	145 (36.9%)	15 (41.2%)
IV	54 (12.6%)	51 (13%)	3 (8.8%)

* , statistical significance (p<0.05).

GOLD; the **G**lobal initiative for **C**hronic **O**bstructive **L**ung **D**isease.

Stage I: FEV_1_ ≧ 80%, Stage II: 50%≦ FEV_1_<80%.

Stage III: 30%≦ FEV_1_<50%, Stage IV: FEV_1_<30%.

PAD, peripheral arterial disease; BW, body weight; BMI, body mass index; DM, diabetes mellitus; PWV, pulse wave velocity; ABI; ankle brachial index.

**Table 2:** Lung function, age and co-morbidities between each COPD grade.

	GOLDStage I	GOLDStage II	GOLDStage III	GOLDStage IV
Participants (n)	n = 28	n = 186	n = 159	n = 54
Age (years)	72.1±8	70.1±10	70.8±8	66.5±10
Pre-bronchodilator				
FEV_1_ (%predicted)	87.8±6.8	62.5±8.2	40.5±5.8	24.3±4.2
FEV_1_/FVC	67.3±3.2	61.9±5.9	53.3±7.5	43.5±7.1
Post-bronchodilator				
FEV_1_ (L)	2.05±0.35	1.54±0.41	1.03±0.26	0.69±0.19
FEV_1_/FVC	67.0±2.3	62.1±6.0	53.7±7.5	43.4±7.6
PAD	2 (7.1%)	15 (8.1%)	14 (8.8%)	3 (5.6%)
Diabetes	4 (14.3%)	34 (18.3%)	29 (18.2%)	11 (20.4%)
Hypertension	15 (53.6%)	82 (44.1%)	83 (52.2%)	27 (50.0%)
Hyperlipidemia	3 (10.7%)	29 (15.6%)	18 (11.3%)	7 (13.0%)

### Prevalence of PAD and risk factors in the elderly COPD patients

In total, 118 patients were aged <65 years (younger COPD patients) and 309 were aged ≥65 years (elderly COPD patients). The prevalence of asymptomatic PAD was 2.5% in the younger COPD patients and 10% in the elderly COPD patients. Because of the small number of asymptomatic PAD subjects (n = 3) in the younger age group, we did not do the analysis of this group. As shown in Table 3, among the elderly COPD patients (n = 309), those with asymptomatic PAD were also older (77.5±6.1 vs. 74.5±5.4 years) and had significantly higher rates of hyperlipidemia (48.4% vs. 9.7%) and hypertension (77.4% vs. 50.7%) than those without asymptomatic PAD (p<0.05). In both the younger and elderly COPD patients, those with asymptomatic PAD had higher rates of diabetes, however the difference did not reach statistical significance. In addition, there was no significant difference in lung function (FEV_1_ and FVC % predicted) between the two groups regardless of age.

**Table 3:** PAD and risk factors in the older COPD patients.

	Age≥65 years (n = 309)	
	PAD (−)	PAD (+)
Participants (n)	278 (90%)	31 (10%)
Age (years, mean)	74.5±5.4	77.5±6.1*
Male gender	272 (97.8%)	31 (100%)
Smoking	508 (62%)	53 (70.7%)
Current	126 (45.3%)	14 (45.2%)
Former	152 (54.7%)	17 (54.8%)
DM	55 (19.8%)	9 (29%)
Hypertension	141 (50.7%)	24 (77.4%)*
Hyperlipidemia	27 (9.7%)	15 (48.4%)*
BMI	22.8±3.2	22.8±3.2
Normal	210 (75.5%)	23 (74.2%)
Overweight	61 (21.9%)	7 (22.6%)
Obese	7 (2.5%)	1 (3.2%)
PWV		
Right	17.3±3.7	16.1±5.6
Left	17.2±4.0	16.8±6.8
ABI	1.08±0.1	0.83±0.2*
FEV_1_ (pred %)	51.8±17.4	53.0±17.8
FVC (pred %)	60.4±15.6	59.5±15.7

* , statistical significance (p<0.05).

PAD, peripheral arterial disease; BMI, body mass index; DM, diabetes mellitus; PWV, pulse wave velocity.

### Independent risk factors associated with PAD in patients with COPD

Multivariate logistic regression analysis revealed potential risk factors for asymptomatic PAD in the enrolled 427 COPD patients. Hyperlipidemia was the strongest independent factor for the development of asymptomatic PAD (odds ratio (OR): 6.89, p<0.001, 95% confidence interval 3.06–15.53). In addition, age≥65 years (OR: 4.80), hypertension (OR: 3.39), and smoking burden (pack-years, OR: 1.02) also reached statistical significance (Table 4).

**Table 4:** Multivariate logistic regression analysis of the factors associated with asymptomatic peripheral arterial disease in all 427 enrolled COPD patients.

Variables	*p* value	Odds ratio	95% confidence interval	
			Lower	Upper
Hyperlipidemia	<0.001	6.89	3.06	15.53
Age≥65 years	0.018	4.80	1.30	17.67
Hypertension	0.009	3.39	1.36	8.47
Pack-years	0.001	1.02	1.01	1.02

## Discussion

To the best of our knowledge, this is the first cross-sectional study to investigate the prevalence of asymptomatic PAD among COPD patients in Asia. We used an ABI<0.9 for the diagnosis of PAD.

Among the enrolled 427 COPD patients, the prevalence of asymptomatic PAD was 8%. The prevalence of asymptomatic PAD was 2.5% in the younger COPD patients and 10% in the COPD patients. Age, hyperlipidemia, and hypertension were the associated factors in the elderly COPD patients. There was no significant difference in lung function (FEV_1_ and FVC % predicted) between the COPD patients with and without PAD. In multivariate logistic analysis, hyperlipidemia was the strongest independent factor for asymptomatic PAD in the patients with COPD.

In a review of the literature, only a few studies were found that investigated the prevalence of asymptomatic PAD in patients with COPD (Table 5). The first study regarding the prevalence of PAD in patients with COPD was in France, which enrolled 151 moderate to severe COPD patients and found that the prevalence of low ABI (<0.9) was 81.4% [16]. This is very high compared to studies from Israel and Spain which reported the prevalence of low ABI to be approximately 30% to 40% [17], [18]. In our study, the prevalence rate was only 8%, which is considerably lower than the previous studies. This is probably because the prevalence of cardiovascular risk factors in our study was much lower than in the previous studies. As shown in Table 5, the prevalence of hyperlipidemia in two of the studies was about 68%, which is almost 5 times higher than in our study (13.3%). Hyperlipidemia was the strongest independent factor for the development of asymptomatic PAD in the current study, and this may explain the low prevalence rate. In addition, the prevalence rates of hypertension, diabetes and obesity in our study were much lower than in previous reports.

**Table 5:** Prevalence and risk factors for PAD in the COPD patients by ethnicity.

Country	Prevalence (%)	Diagnostic method	Enrolled subjects	Lung function	Hyperlipidemia	Diabetes	Hypertension	Others
France [16]	81.4	ABI<0.9	151 moderate-to-severe COPD patients, mean age: 67±3.1 years	FEV1: 37±6 %FEV1/FVC :47±4.3 %	68.1%	25.6%	74.8%	BMI: 22.9
Israel [17]	31	ABI<0.9	87 COPD patients, mean age: 69.8±11.8 years	FEV1: 34±8%, with PADFEV1:45±16 %, without PAD	Unavailable	43 %	72.4%	BMI: 29.3
Spain [18]	36.8	ABI<0.9	246 COPD patients, mean age: 70.2±11 years; men: 79%	FEV1: 46±15 %, with PADFEV1:52±14 %, without PAD	68%	27.2%	57.3%	Obesity: 33%
Taiwan	8	ABI<0.9	427 COPD patients, mean age: 70.0±9 years; men: 97.7%	FEV1: 51.2±18 %FVC :62.2±17 %	13.3%	18.3%	48.5%	BMI: 23Obesity: 3.3%

ABI, ankle-brachial index; PAD, peripheral arterial disease.

Several studies have shown that reduced pulmonary function is independently associated with subclinical atherosclerosis, arterial stiffness and coronary heart diseases [24]–[28]. Most of the results showed that PWV was significantly and negatively associated with FEV_1_ and FVC [24], [25]. However, few studies have investigated the relationship between ABI<0.9 and lung function. Two studies found that COPD patients with PAD had a worse lung function [17], [18], however, there were statistical limitations to both of these studies. In the study from Israel, multivariate regression analysis was not used to adjust for confounding factors [17]. In the study from Spain, COPD severity was found to be positively associated with ABI<0.9, however, they did not consider cardiovascular factors such as diabetes, hyperlipidemia, and hypertension as co-variables for multivariate regression analysis [18]. In our study, we did not find any association between lung function and low ABI (<0.9), in either the younger or elderly COPD patients. However, this is the only study to report a negative association between asymptomatic PAD and lung function, and further large-scale investigations are necessary to confirm this finding.

According to our findings, the slightly higher prevalence rate of asymptomatic PAD among COPD patients than in the general population may be due to smoking. As in previous reports, smoking is the most powerful modifiable vascular risk factor for the development of PAD [29]. However, people often find it difficult to stop smoking before they become ill, and this is probably the reason why the prevalence of current smokers (67% vs. 45%) was higher in the younger than in the elderly patients in our study.

A greater BMI is associated with the development of PAD [30]–[33]. However, patients with COPD tend to be underweight and cachexic. The mechanism underlying this phenomenon involves systemic inflammation and impaired muscle oxidation [34]–[36]. In our study, only 3.7% of all enrolled COPD patients were obese, which is much lower than our previous data for the general population (overweight: 31.9% and obese: 11.2%) [37]. Thus, COPD related cachexia may be a protective effect against the development of PAD.

There are some limitations to this study. First, most of the enrolled patients were male (97.5%), thus, it is unknown whether the results can be applied to women. Second, we did not use the 2011 revision of the GOLD guidelines for COPD severity classification which have been modified by the inclusion of dyspnea scores. However, a direct comparison with lung function can provide more objective results.

In conclusion, the prevalence of asymptomatic PAD was 8% in COPD patients (2.5% in the younger patients and 10% in the elderly patients). Hyperlipidemia was the strongest independent factor associated with asymptomatic PAD, followed by old age and hypertension. Lung function was not associated with PAD in the patients with COPD.
